# Comparative efficacy of tetracyclines against isolates of *Mycobacterium avium* complex

**DOI:** 10.5588/ijtldopen.24.0551

**Published:** 2025-02-01

**Authors:** S. Singh, G.D. Boorgula, A. Shrivastava, T. Gumbo, S. Srivastava

**Affiliations:** ^1^ Division of Infectious Diseases, Department of Medicine, School of Medicine, University of Texas at Tyler, Tyler, TX, USA;; ^2^ Mathematical Modeling and AI Department, Praedicare Inc., Dallas, TX, USA;; ^3^ Hollow Fiber System & Experimental Therapeutics Laboratories, Praedicare Inc., Dallas, TX, USA;; ^4^ Department of Cellular and Molecular Biology, University of Texas Health Science Centre at Tyler, Tyler, TX, USA.

**Keywords:** nontuberculous mycobacteria, *M. avium* complex, MIC, MAC-PD

Dear Editor,

The standard guideline-based treatment (GBT) for *Mycobacterium avium* complex (MAC) pulmonary disease (MAC-PD) is a combination of macrolide (clarithromycin or azithromycin) plus ethambutol and a rifamycin (rifabutin or rifampin). Treatment can last for up to 18 months,^1^ with sustained sputum culture conversion (SSCC) rates ranging between 35% and 53%, and adverse reactions are not uncommon.^2-4^ Therefore, the search continues for better drugs with improved efficacy and lower toxicity. Tetracycline, discovered in the late 1940s, inhibits bacterial protein synthesis, specifically preventing the association of aminoacyl-tRNA with the bacterial ribosome.^5,6^ There are reports on the efficacy of tetracyclines against MAC based on in vitro preclinical pharmacokinetic/pharmacodynamics (PK/PD) studies on minocycline and its newer congeners, tigecycline and omadacycline, as well as sarecycline.^7–10^ There is also at least one clinical study that demonstrated the efficacy of minocycline in combination therapy.^11^ Here, we compared different generations of tetracyclines (oxytetracycline, tigecycline, minocycline, omadacycline, and eravacycline) minimum inhibitory concentrations (MIC) and static concentration-response studies against the reference laboratory strain (American Type Culture Collection [ATCC] #700898) and 44 de-identified clinical isolates (*M. avium* subsp *avium*: *n* = 15; *M. avium* subsp *intracellulare*: *n* = 24; and *M. avium* subsp *chimaera*: *n* = 5). This is an initial step in ranking the tetracyclines for potential PK/PD studies in treating MAC-PD.

The Clinical and Laboratory Standards Institute (CLSI) recommended broth microdilution method be used for MIC testing using cation-adjusted Mueller-Hinton broth supplemented with 5% oleic acid, albumin, dextrose and catalase (OADC) and for inoculum preparation.^12^ Drugs were purchased from BOC Sciences (New York, NY, USA) and the University of Texas Health Science Center at Tyler campus pharmacy. The drug concentrations ranged from 0.06 mg/L to 64 mg/L, except for eravacycline, which ranged from 0.03 mg/L to 20 mg/L. Since omadacycline degrades in solution, we used 0.5% (v/v) oxyrase to prevent drug degradation.^13^ The cultures were incubated at 37^0^C for 7 days before the MICs were read using an inverted mirror. All experiments were performed in duplicate and repeated twice. MS Excel (Microsoft, Seattle, WA, USA) was used to record the data and MIC values for the cumulative percentage of the isolates, i.e., MIC_50_ and MIC_90_. No ethical approval was needed for this study.

The MIC of the reference laboratory strain, ATCC#700898, against oxytetracycline was 128 mg/L, for tigecycline this was 1 mg/L, for minocycline 2 mg/L, for omadacycline (with 0.5% v/v oxyrase) 0.5 mg/L, and for eravacycline 0.03 mg/L. The MIC distribution for each of the five antibiotics against MAC clinical strains is shown in Table 1. The MIC_50_ and MIC_90_ for tetracycline, tigecycline, and omadacycline were >64 mg/L. Minocycline MIC_50_ was 32 mg/L, and MIC_90_ was calculated as 64 mg/L. For eravacycline, both MIC_50_ and MIC_90_ were calculated to be >20 mg/L. In agreement with previous reports, oxyrase did not improve the MIC of tigecycline^14^ or tetracycline. However, a one-tube dilution shift in minocycline MICs was observed that resulted in MIC_50_ changing from 32 mg/L to 16 mg/L, but the MIC_90_ remained at 64 mg/L (data not shown). The static concentration-response studies with the standard reference strain used Middlebrook 7H9, supplemented with 10% OADC; Middlebrook 7H10 agar, supplemented with 10% OADC, was used for colony-forming unit (CFU) estimation. For CFU estimations, the experiments were conducted in a total volume of 5 mL in screw-capped tubes at the drug concentration range mentioned above. After 7 days of co-incubation, 1 mL cultures were collected in sterile centrifuge tubes and washed with normal saline twice to remove the carry-over drug. The bacterial pellet was resuspended in normal saline, and 10-fold serial dilutions were prepared, followed by spreading the cultures on 7H10 agar. Cultures were sealed in a Ziploc bag and incubated at 37^0^C for 10 days before the CFUs were recorded. Data were analyzed using the inhibitory sigmoid maximal effect (E_max_) model (GraphPad Prism v10; GraphPad Software, La Jolla, USA). The kill below stasis (Day 0 bacterial burden) with each drug (mean ± standard deviation) and the concentration mediating 50% of maximal effect (EC_50_) with each drug are summarized in Table 2, showing all five tested tetracyclines killed the MAC reference strain.

**Table 1.** Comparison of MICs against 44 *Mycobacterium avium* clinical isolates.

SN	Subsp.	Oxytetracycline MIC mg/L	Tigecycline MIC mg/L	Minocycline MIC mg/L	Omadacycline MIC mg/L	Eravacycline MIC mg/L
1	*M. avium*	0.5	1	0.5	1	>20
2	*M. avium*	4	8	2	4	>20
3	*M. avium*	>64	>64	32	16	>20
4	*M. avium*	64	64	16	64	10
5	*M. avium*	>64	>64	32	>64	20
6	*M. avium*	32	32	8	64	20
7	*M. avium*	64	32	4	2	>20
8	*M. intracellulare*	>64	>64	32	>64	>20
9	*M. intracellulare*	>64	>64	64	>64	>20
10	*M. intracellulare*	>64	>64	64	32	>20
11	*M. intracellulare*	>64	>64	64	64	>20
12	*M. intracellulare*	>64	>64	64	>64	>20
13	*M. intracellulare*	>64	>64	32	64	>20
14	*M. intracellulare*	>64	>64	64	64	20
15	*M. chimaera*	>64	64	64	64	>20
16	*M. chimaera*	>64	8	32	>64	>20
17	*M. chimaera*	>64	>64	32	32	>20
18	*M. chimaera*	>64	>64	>64	32	>20
19	*M. intracellulare*	>64	>64	32	>64	>20
20	*M. intracellulare*	>64	>64	>64	64	>20
21	*M. intracellulare*	>64	>64	>64	32	>20
22	*M. intracellulare*	>64	64	>64	16	>20
23	*M. intracellulare*	0.25	0.25	0.25	0.25	20
24	*M. intracellulare*	>64	64	64	16	20
25	*M. intracellulare*	>64	>64	>64	32	>20
26	*M. intracellulare*	64	16	64	2	20
27	*M. intracellulare*	>64	>64	>64	64	20
28	*M. avium*	>64	0.5	>64	4	20
29	*M. intracellulare*	4	8	4	32	2.5
30	*M. avium*	64	64	16	64	10
31	*M. chimaera*	64	64	8	>64	20
32	*M. avium*	>64	>64	16	>64	>20
33	*M. avium*	32	64	16	>64	>20
34	*M. intracellulare*	>64	64	64	>64	>20
35	*M. intracellulare*	>64	>64	64	64	>20
36	*M. intracellulare*	64	>64	32	64	>20
37	*M. intracellulare*	>64	>64	64	64	>20
38	*M. avium*	32	16	16	64	10
39	*M. intracellulare*	>64	>64	32	64	>20
40	*M. intracellulare*	64	16	8	32	10
41	*M. avium*	>64	>64	32	64	10
42	*M. avium*	16	4	2	4	10
43	*M. intracellulare*	>64	>64	32	64	>20
44	*M. avium*	16	8	8	64	20
	MIC_50_	>64	>64	32	>64	>20
	MIC_90_	>64	>64	64	>64	>20

MIC = minimum inhibitory concentration.

**Table 2.** Comparison of five tetracyclines for anti-MAC activity.

	Oxytetracycline	Tigecycline	Minocycline	Omadacycline	Eravacycline
Kill below stasis,^*^ log_10_ CFU/mL	5.25 + 0.07	3.93 + 0.33	1.59 + 0.24	2.29 + 0.16	4.81 + 0.79
EC_50_, mg/L	4.06 + 5.05	0.01 + 0.05	0.76 + 0.09	8.44 + 1.95	0.28 + 0.12

* Stasis or bacterial burden in the inoculum.

MAC = *M. avium* complex; CFU = colony-forming unit; EC_50_ = drug concentration mediating 50% of the maximal effect calculated using the inhibitory sigmoid maximal effect model.

The MIC data only have meaning when compared to concentrations of each drug achieved in lung lesions of patients, and the tolerability of drugs (e.g., tigecycline versus omadacycline) and the tolerability of doses that achieve those concentrations. Moreover, highly degraded drugs (such as omadacycline) will demonstrate high EC_50_ if drug degradation is not accounted for. In this case, it is instructive that in preclinical studies of the hollow fiber system of MAC lung disease (HFS-MAC) in which the drug was administered fresh each day, the best kill below stasis was with omadacycline at 4.86 log_10_ CFU/mL, which was similar to tigecycline, followed by minocycline at 3.60 log_10_ CFU/mL, whereas sarecycline 1.0 log_10_ CFU/mL was least effective.^8–10,15^ The discrepancy with static concentration-effect studies is because HFS-MAC has a fresh drug each day, the concentration-time profiles in HFS-MAC decay similar to those in patient lesions, and MIC is not a very good surrogate of the efficacy of tetracyclines against slow-growing mycobacteria such as MAC.^7^

To summarize, there is a lack of effective treatment options for MAC-PD. Given the anti-MAC activity of tetracyclines, these drugs should be carefully tested at the dynamic concentration in pre-clinical models to combine with other effective drugs to develop better regimens for MAC-PD.
